# The Influence of Different Types of Trust, Perceived Objectivity, and Perceived Risk on the Preferred Involvement of AI for Subjective and Objective Tasks

**DOI:** 10.1111/risa.70349

**Published:** 2026-09-15

**Authors:** Fabienne Michel, Michael Siegrist

**Affiliations:** ^1^ ETH Zurich, Institute for Environmental Decisions (IED), Consumer Behavior Zurich Switzerland

**Keywords:** AI acceptance, AI confidence, AI trust, general confidence, general trust, psychometric paradigm, social trust

## Abstract

Artificial intelligence (AI) is increasingly involved in decision‐making. While many consumers currently use AI for minor tasks such as music recommendations or for improving writing, it can also be used for more consequential decisions such as medical diagnoses or financial recommendations. A declaration of AI content is not common, and consumers may not always be aware that AI is involved in the decision‐making. Therefore, it is important to understand their perception of AI for different tasks and how different kinds of trust influence the degree to which AI involvement is accepted. The psychometric paradigm was adapted to analyze participants’ acceptance, perceived objectivity, and perceived risks in the event of errors for a list of different tasks. To analyze the role of trust, measures for general confidence, general trust, and social trust, as well as AI‐trust and AI‐distrust were collected. The online survey was completed by 923 Swiss participants. The results show that participants’ preferred levels of AI involvement were influenced positively by the degree of subjectivity and negatively by the perceived risk associated with the task. Preferred AI involvement was highest for “Searching for information,” “Making a weather forecast,” and “Programming software” but lowest for “Delivering a court judgment,” “Selecting a candidate for a job,” and “Driving a car.” General confidence, general trust, and social trust indirectly influenced acceptance of AI through AI‐trust, but not AI‐distrust. The findings indicate that although specific AI‐trust has the largest influence on acceptance of AI, general types of trust are also important for evaluating AI in decision‐making.

## 1 Introduction

Decisions made under uncertainty often rely on advice from experts—but what happens when the expert is not a human but an artificial intelligence (AI)? Such situations become more likely as AI plays an increasingly prominent role in decision‐making across various areas of life, often without one's awareness. Although many AI applications assist with everyday tasks, such as recommending music or improving writing, some are used to make more consequential decisions such as selecting candidates for a job or determining a person's creditworthiness. Currently, AI provides recommendations, but humans make the final decision in most cases. In the future, even more decisions may be delegated to AI. In this article, we therefore address two complementary research aims related to risk perception and acceptance of AI. The first research aim builds on the psychometric paradigm and relates to the qualitative characteristics of a task that influence acceptance of AI in decision‐making. The second research aim focuses on the influence of trust and confidence on the preferred involvement of AI and addresses the role of general trust‐related measures such as social trust, general trust, and general confidence as antecedents.

### 1.1 The Psychometric Paradigm

The psychometric paradigm has often been used to study how lay people perceive various hazards (Siegrist et al. 2008; Slovic 1987) and helps to explain why different hazards are perceived differently. Generally, participants are asked to assess a hazard on various aspects. Related to the perception of technological risks, participants have been rating perceived dreadfulness or how much control they have over their exposure to a hazard (Slovic et al. 1986). Although AI may not necessarily be perceived as a hazard, the psychometric paradigm nevertheless offers a valuable tool to better understand how potential risks, such as AI applications, are perceived in regard to different aspects. Building on the findings of previous studies in the field of AI and algorithmic perception (Castelo et al. 2019; Grassini et al. 2025), the aspects included in this study are the degree of subjectivity versus objectivity and the perception of risks in the event of errors.

### 1.2 Areas Where AI Currently Is and Is Not Accepted

Previous research has shown that people are often reluctant to rely on algorithms for decision‐making (Castelo et al. 2019; Dietvorst et al. 2015). This phenomenon is called algorithm aversion and has been demonstrated in various decision settings such as financial risk management (Larkin et al. 2021) or health care (Kerstan et al. 2024; Larkin et al. 2021; Longoni et al. 2019; Promberger and Baron 2006). Especially in medical settings, people strongly prefer to receive medical advice from human doctors compared to algorithms (Larkin et al. 2021; Promberger and Baron 2006) and are hesitant to engage with healthcare services offered by AI, both in real‐life situations and hypothetical scenarios (Longoni et al. 2019).

AI applications will never be flawless, and the chance of a bad decision will always be present. When consumers realize that algorithms make mistakes, some studies show that their confidence in them declines, and that they are more likely to reject an algorithm than a human for making the same mistake (Dietvorst et al. 2015). However, perceived risk does not necessarily reduce the acceptance or trust toward AI (Grassini et al. 2025).

There are domains in which people may prefer algorithmic over human advice, such as for weather forecasts, data analysis, navigation, or predicting stock markets as participants of an online survey indicated higher trust in algorithms than in humans for these tasks (Castelo et al. 2019). In another study, where participants were acting as investors and chose either a human fund manager or an investment algorithm, participants showed no algorithm aversion (Germann and Merkle 2022). The authors attribute this finding to people's lack of firm preferences and that participants were primarily concerned with returns. They also note that their participants did not lose confidence in the algorithm after seeing it make a mistake (Germann and Merkle 2022).

It has been suggested that people's willingness to accept AI depends on the decision‐making context (Castelo et al. 2019; Grassini et al. 2025). For example, the use of AI for traffic control was rated more favorable than the use of AI for personalized product recommendations (Grassini et al. 2025). It seems that people view AI as most reliable for tasks that involve evaluating large amounts of data and modeling such data, as these are areas typically viewed as strengths of machines and computers (Grassini et al. 2025).

Although people often see objective tasks as requiring careful reasoning and systematic evaluation, they perceive subjective tasks as depending more on personal judgement and gut feeling (Inbar et al. 2010). This influences how appropriate or acceptable the use of AI is considered for different tasks. For a numerical task, where there is only one objectively correct answer, participants relied more often on algorithms than humans (Logg et al. 2019). In another study, participants rated humans to be better than an algorithm for a subjective task like predicting how funny a joke is (Yeomans et al. 2019). Castelo et al. (2019) presented participants with a variety of tasks, including hiring and separation of employees, driving a subway, investing in stocks, and playing the piano. In a series of studies, participants assessed the degree of subjectivity versus objectivity, trust in humans versus algorithms, and preference for human versus algorithmic performance across these tasks. The results indicated that, on average, participants had more trust in a qualified human than in algorithms. However, a higher preference level of algorithms than humans was found for specific tasks like stock market predictions, weather forecasts, data analysis, and providing directions (Castelo et al. 2019). The study of Castelo et al. (2019) further indicated that algorithms were less trusted and used for tasks considered subjective. The authors showed that this observed effect occurred primarily due to the prevailing perception that algorithms are ineffective at subjective tasks.

### 1.3 Trust Explaining Individual Differences in Acceptance of AI

Trust and confidence are important factors that influence people's perceptions of risk and their acceptance of novel technologies (Siegrist 2021). Accordingly, many studies have examined the role of trust on the acceptance of AI as a decision‐support tool (Duan et al. 2025; Fahnenstich et al. 2024; Kerstan et al. 2024; Liu and Tao 2022; Liu et al. 2019; Mayer et al. 2024; Molina and Sundar 2024). Considerable differences exist between the studies regarding the theoretical conceptualization of trust and how trust has been measured. Liu et al. (2019) conducted a study examining public acceptance of fully automated driving. Their path model suggests that social trust influences the perceived benefits and risks of this technology, as well as acceptance of it. The social trust measure used in that study was related to trust in the government agencies that regulate automated driving and the companies that produce such vehicles. In a study by Kerstan et al. (2024), which examined preferences for human versus AI doctors, trust was measured using semantic differential items such as “useful” versus “useless,” “unhelpful” versus “helpful,” and “untrustworthy” versus “trustworthy.” This construct was shown to influence how people viewed the risks and benefits of AI in healthcare. Even though this construct has been labeled “explicit comparative trust associations” (Kerstan et al. 2024), it seems to be more closely related to the concept of attitudes than to a specific measure of trust (Siegrist 2021). In a study by Molina and Sundar (2024), dispositional trust, also known as general trust in humans, was used to explain individual differences in trust regarding AI. Thus, past studies examining trust and acceptance of AI suggest that trust is important. However, these studies lack insight into which type of trust is most important and whether different types of trust have additive or opposing effects. Research on risk perception related to the coronavirus (SARS‐CoV‐2), for example, has shown that opposing effects are possible (Siegrist, Luchsinger, et al. 2021).

**FIGURE 1:**
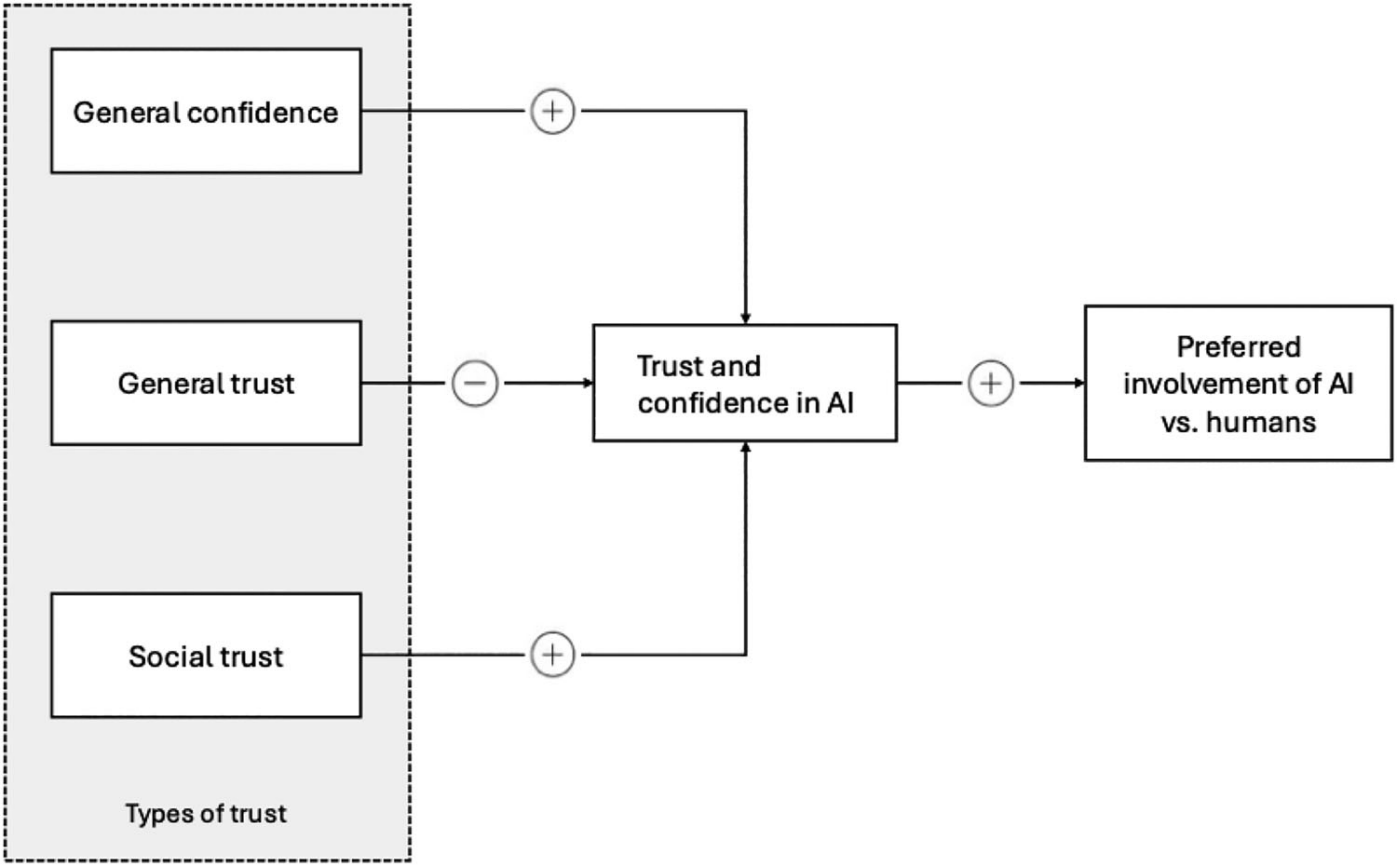
Model of postulated effects and expected positive or negative association between general types of trust as well as trust and confidence in AI on preferred involvement of AI versus humans for different tasks.

We postulate that the preferred level of involvement of AI across different tasks is influenced by specific trust and confidence in AI. Trust and confidence in AI, in turn, will be influenced by more general forms of trust, such as general confidence, general trust, and social trust. To facilitate the presentation of our theoretical model (see Figure 1), we first describe the more general trust‐related constructs before introducing the AI‐specific measures.

General confidence is a broad construct. It is the belief that events will occur as expected in the future, that uncertainty will be low, and that society will be able to cope with future problems and risks (Keller et al. 2011). Studies have found that general confidence is negatively related to various risk perceptions (Keller et al. 2011; Siegrist, Luchsinger, et al. 2021). This means that people with high general confidence perceive a lower level of risk from various hazards than people with low general confidence. It thus seems reasonable to assume that people who generally have a high general confidence would also show more trust and confidence in a specific technology, such as AI. Therefore, we expected general confidence to positively influence trust and confidence in AI, which would, in turn, positively influence the preferred involvement of AI for different tasks.

General trust is a personality trait that measures how much one trusts people met for the first time. Researchers have found that general trust is often negatively correlated with risk perceptions across a broad range of hazards (Siegrist et al. 2005; Siegrist, Luchsinger, et al. 2021). In the case of COVID‐19, general trust reduced perceived risk, however (Siegrist and Bearth 2021). This could be similar in the case of AI. As AI can function autonomously and potentially replace human decision‐makers, it seems plausible that high general trust in people could hinder this idea of replacing human decision‐makers with AI. Conversely, people with low general trust could be more open to replacing human decision‐makers with AI, as they might see AI as more reliable and less biased than humans. For these reasons, we expected general trust to negatively affect trust and confidence in AI and that this would further result in lower preferred involvement of AI for different tasks. In other words, people who tend to trust other people unconditionally are less likely to have trust and confidence in AI and to prefer the involvement AI for different tasks than people who show less trust in other people.

Finally, social trust has also been found to be important for the perception of many hazards (see Siegrist 2021), and it has also been shown to be important in the case of AI (Liu et al. 2019). Social trust refers to the willingness to rely on those who oversee and manage a certain field (Siegrist et al. 2000). People tend to trust institutions whose values align with their own, and this trust serves as a heuristic cue for evaluating risks and acceptance of a technology (Siegrist 2021). We hypothesized that people's trust and confidence in AI are positively influenced by their social trust in authorities regulating AI and overseeing companies providing AI services, and that this leads to a higher preferred involvement of AI compared to humans for specific applications.

### 1.4 Aims

The present study first aimed to examine the perception of AI for different tasks in order to identify factors that suggest higher or lower preferred involvement of AI. To address this, we used an adapted version of the psychometric paradigm to investigate the influence of perceived objectivity and perceived risk in the event of errors on the preferred involvement of AI.

The second aim of the study was to examine how different types of trust influence people's perception of AI, as well as whether these types of trust directly and indirectly influence the preference of AI over humans for different tasks. Our proposed model is shown in Figure 1. We expect that a measure of trust and confidence specifically in AI will influence the preferred level of involvement of AI for different tasks and that this measure is influenced by general confidence, general trust, and social trust. Trust and confidence in AI will be important for the acceptance of AI because, like humans, AI is expected to give reliable recommendations and to not make mistakes.

## 2 Methods

### 2.1 Participants

Participants were recruited in the German‐speaking part of Switzerland by a commercial panel provider (Bilendi Schweiz AG, Zurich). Quota for age (20–29, 30–39, 40–49, 50–59, 60–69) and gender were set to ensure a balanced sample. Complete data from 1106 participants were collected. Thereof, 183 participants were removed from the data set because they did not correctly answer the attention check question. The attention check question asked participants to choose the answering option “fully agree” and was positioned as an item of a scale. The final dataset comprises the answers of 923 Swiss participants (50.6% female, 48.8% males, 0.6% non‐binary/not indicated). The mean age was 45 years (SD = 14).

### 2.2 Questionnaire

The survey was conducted in German and programmed using the survey platform Question Pro (Question Pro Inc., Austin, TX, USA). Data collection took place in March 2025. After indicating socio‐demographic data such as age, sex, and education, participants were presented with a list of tasks. Building on the list of tasks used in the research of Castelo et al. (2019), the tasks for the list were carefully chosen to reflect what AI is already used for and what AI could be used for in the (near) future, but also to represent different skill sets (analytical, creative) needed to complete the tasks, as well as to illustrate different levels of outcome consequences. Examples of these tasks are “Selecting a candidate for a job” or “Writing a non‐fiction book.” For the full list, please refer to Table 1.

**TABLE 1:** Preferred involvement of artificial intelligence (AI), perceived objectivity, and perceived level of risk for selected tasks (*N* = 923).

	Preferred involvement of AI		No. of participants preferring 0% AI	Perceived objectivity		Perceived level of risk	
Item	Mean	SD	No. (%)	Mean	SD	Mean	SD
Searching for information	66.78	24.44	21 (2.3)	59.28	24.77	40.23	23.82
Making a weather forecast	62.02	25.96	38 (4.1)	63.00	25.28	41.16	24.15
Programming software	61.85	25.43	38 (4.1)	62.39	26.65	52.15	23.98
Grading a math exam in high school	48.85	30.95	89 (9.6)	68.72	26.97	41.67	25.14
Planning a financial investment that fits a risk preference	47.76	26.61	66 (7.2)	52.75	25.23	63.46	21.16
Writing a nonfiction book	47.34	28.63	70 (7.6)	62.48	25.50	41.54	24.31
Determining a person's creditworthiness	41.43	28.86	102 (11.1)	62.21	25.38	56.02	22.90
Investing your pension fund's money	39.70	26.67	90 (9.8)	58.25	24.13	64.50	22.13
Writing a newspaper article	39.43	26.68	91 (9.9)	50.96	23.87	43.24	24.19
Grading an essay in high school	33.22	26.47	127 (13.8)	51.30	24.88	43.80	23.98
Composing a song	32.78	27.83	153 (16.6)	33.41	26.36	25.29	24.67
Making a medical diagnosis	32.73	24.85	119 (12.9)	65.33	24.48	72.32	22.84
Selecting a medical treatment	31.14	24.73	135 (14.6)	59.18	24.46	70.77	23.27
Writing a novel	28.60	26.21	157 (17.0)	34.22	26.06	27.76	24.40
Driving a car	28.48	27.93	194 (21.0)	59.60	27.16	70.78	24.57
Selecting a candidate for a job	25.89	24.23	169 (18.3)	51.72	24.13	52.82	22.01
Delivering a court judgment	21.57	23.45	219 (23.7)	62.98	24.13	71.12	23.21

*Note*: Preferred involvement of AI was measured as preferred level of AI over human involvement from 0 = “100% human” to 100 = “100% AI.” Perceived objectivity was measured from 0 = “completely subjective” to 100 = “completely objective.” Perceived level of risk was measured from 0 = “very low risk” to 100 = “very high risk.”

Participants were asked to indicate how subjective or objective they perceived each of these tasks to be. Tasks were displayed in random order, and answers were given by moving a slider on a scale ranging from 0 to 100 (numbers not shown) that was only verbally anchored from “completely subjective” to “completely objective.” Next, participants were presented with a definition of AI. The definition read, “Artificial Intelligence (AI) describes particularly advanced technical systems. AI performs tasks that usually require human intelligence, such as visual perception, speech recognition, and decision‐making” and was originally proposed and used by Kerstan et al. (2024). Participants were reassured that we were interested in their opinion and that there were no right or wrong answers.

Next, participants were asked to rate the degree to which a human or an AI should be involved for the same tasks that they already evaluated regarding perceived objectivity. Again, tasks were presented in random order. Participants indicated their answer by shifting a slider to the desired position on a scale from 0 to 100. The endpoints were only verbally anchored “100% human (0% AI)” and “100% AI (0% human).” This measure is being referred to it as *preferred involvement of AI* throughout the manuscript to avoid overly lengthy phrasing. Afterward, participants were again presented with a randomized order of the same tasks but this time asked to indicate how high they perceived the risks to be in case of errors. Answers were collected on a scale from 0 to 100, and again only verbally anchored “very low risk” and “very high risk.”

*General confidence* was assessed using the six‐item scale by Keller et al. (2011). This scale includes items such as “In the future, society will be functioning as well as today” and “Altogether, we live in a safe and secure time.” Participants indicated their answers on a 7‐point Likert scale from “1 = do not agree at all” to “7 = fully agree.” Again, only endpoints were verbally labeled. The scale showed a high internal consistency (Cronbach's *α* = 0.87).

To assess *general trust*, the six‐item general trust scale by Siegrist et al. (2005) was used, which is based on items from previous literature (Glaeser et al. 2000; Rotter 1967; Yamagishi 1988). Example items are “If given a chance, most people would try to take advantage of you” and “You can't trust strangers anymore” (Siegrist et al. 2005). Answers were collected on a 7‐point Likert scale from “1 = do not agree at all” to “7 = fully agree” whereby only endpoints were verbally labeled. The scale yielded good internal consistency (Cronbach's *α* = 0.83).

Three items to measure *social trust* in industry and regulators were developed ad hoc. This scale consisted of the items “You can trust the information from AI providers,” “I trust the authorities to regulate AI systems,” and “I have confidence in the authorities that the misuse of AI systems will be punished.” The items were measured on a 7‐point Likert scale, where only endpoints were verbally labeled, from “1 = do not agree at all” to “7 = fully agree,” and showed good internal consistency (Cronbach's *α* = 0.80).

“Confidence and trust in AI” was measured using eight self‐developed statements. Examples are “AI can assess the consequences of its actions” and “AI can intentionally do bad things.” Participants were asked to indicate their agreement to these statements on a 7‐point Likert scale from “1 = do not agree at all to 7 = fully agree.” To assess these items, a principal axis factor analysis was conducted. The findings are detailed in Section 3.

The questions presented here were part of a larger research project that also included an unrelated series of experiments. For the sake of clarity, only results related to the perception of AI for the use in different tasks are reported here.

### 2.3 Data Analysis

First, a descriptive analysis on the two aspects, perceived objectivity and perceived level of risk, was performed. To examine whether the preferred level of AI involvement could be predicted by the level of perceived objectivity or the perceived level of risk in the event of error, a regression analysis using the data aggregated at the task level was conducted. Then, it was analyzed how different types of trust would influence people's preferred level of AI involvement, and whether trust and confidence in AI would serve as an intermediary variable. To analyze this mediation model, the procedure explained by Hayes (2022) was followed, and version 5 of the PROCESS macro for SPSS was used. All statistical analyses were performed using IBM SPSS Statistics for Mac, version 29.0 (IBM Corp., Armonk, NY, USA).

## 3 Results

### 3.1 Preferred Involvement of AI

To which degree should a human or an AI be involved in different tasks? The analysis of the mean values for the aggregated data revealed that “Delivering a court judgment” (*M* = 21.57, SD = 23.45) was rated to be the task that is most attributed to humans, whereas “Searching for information” (*M* = 66.78, SD = 24.44) was rated as the task with the highest acceptable involvement of AI. Mean values and standard deviations of the preferred involvement of AI for all 17 items, as well as the number of participants indicating zero preferred involvement of AI, are presented in Table 1. Although the mean preferred involvement of AI varied, it is notable that, on average, participants preferred a larger involvement of humans compared to AI for most tasks but still accepted the involvement of AI to around 20% in all tasks. The percentage of participants who preferred AI not to be involved at all varied between 2.3% for the information search task and 23.7% for delivering a court judgment. This indicates that the proportion of people rejecting any involvement of AI is rather low.

### 3.2 Perceived Objectivity

Participants’ rating of the perceived objectivity versus subjectivity of the different tasks is reported next. Although 15 of the tasks were rated to be rather objective, only two tasks (“Composing a song” and “Writing a novel”) were perceived to be on the subjective side. Mean values and standard deviations are shown in Table 1.

### 3.3 Perceived Level of Risk in the Event of Error

Although perceived objectivity ratings might help to explain the preference of humans over AI for some of the tasks, the perceived level of risk in the event of error might expand the view. Participants associated “Composing a song” (*M* = 25.29, SD = 24.67) and “Writing a novel” (*M* = 27.76, SD = 24.40) with the smallest risks, whereas they indicated “Making a medical diagnosis” (*M* = 72.32, SD = 22.84), “Delivering a court judgment” (*M* = 71.12, SD = 23.21), “Driving a car” (*M* = 70.78, SD = 24.57), as well as “Selecting a medical treatment” (*M* = 70.77, SD = 23.27) to be the tasks with the highest risks. Table 1 shows the mean values and standard deviation for the perceived level of risk in the event of an error for all tasks.

### 3.4 Relationship Between Preferred Involvement of AI, Perceived Objectivity, and Perceived Level of Risk

**FIGURE 2:**
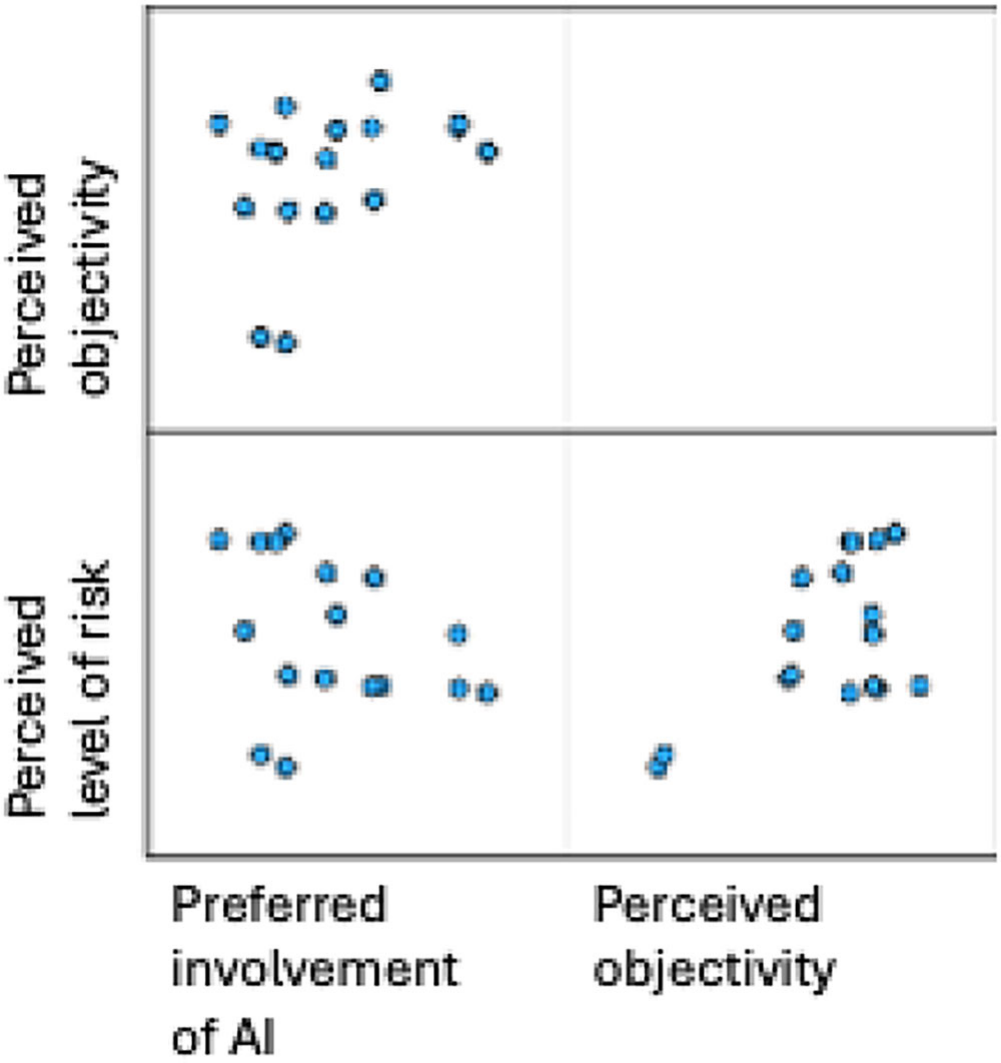
Scatterplot of the correlations between preferred involvement of AI, perceived objectivity, and perceived level of risk for the 17 selected tasks.

To investigate the relationship between preferred involvement of AI, degree of perceived objectivity, and perceived level of risk in the event of error, we first looked at the correlations between the mean values of these aspects for the different tasks. As can be seen in Figure 2, acceptance of AI was associated with perceived objectivity and with perceived level of risk. In addition, perceived objectivity and perceived risk were correlated as well (*N* = 17, *r* = 0.57, *p* = 0.02). In order to examine the impact of perceived objectivity and perceived level of risk on preferred involvement of AI, we conducted a regression analysis. The results indicate that both, perceived objectivity and perceived level of risk, are significant predictors for the preferred involvement of AI, see Table 2.

**TABLE 2:** Regression analysis predicting preferred involvement of AI from perceived objectivity and perceived level of risk.

Predictor	*B*	95% CI	*β*	*t*	*p*
(Intercept)	14.8	[−16.61, 46.26]		1.01	0.329
Perceived objectivity	1.06	[0.39, 1.73]	0.8	3.41	0.004
Perceived level of risk	−0.07	[−1.09, −0.22]	−0.7	−3.30	0.006

*Note: R* ^2^ = 0.50.

### 3.5 Categorization of Different Tasks

To discover whether an underlying structure influenced the preferred involvement of AI for the different tasks, a principal axis factor analysis was performed. The item “Writing a newspaper article” was removed due to high cross‐loadings. The scree plot and the eigenvalue criteria suggested a two‐factor solution. A following quartimax rotation with Kaiser normalization resulted in two factors, explaining 49.3% of the total variance. The factor loadings are depicted in Table 3. The two factors happened to correspond to participant's perceived level of objectivity: Participants’ ratings of perceived objectivity versus subjectivity of the tasks “Composing a song” and “Writing a novel” were below 50, which indicates that these tasks are perceived as subjective (see Table 1). The factor analysis supported this distinction into objective and subjective tasks as the perceived subjective tasks loaded on Factor 2 and all other tasks loaded on Factor 1. As a result, Factor 1 was named “objective tasks,” and Factor 2 was named “subjective tasks.”

**TABLE 3:** Rotated factor loadings for the acceptance of artificial intelligence (AI) (*N* = 923).

Rotated factor loadings	Objective tasks	Subjective tasks
Selecting a candidate for a job	**0.54**	0.16
Composing a song	0.16	**0.69**
Searching for information	**0.48**	0.12
Programming software	**0.54**	0.09
Making a medical diagnosis	**0.71**	−0.04
Selecting a medical treatment	**0.67**	−0.03
Planning a financial investment that fits a risk preference	**0.70**	−0.15
Delivering a court judgment	**0.57**	0.09
Driving a car	**0.60**	−0.13
Making a weather forecast	**0.60**	0.00
Grading a math exam in high school	**0.68**	0.04
Grading an essay in high school	**0.54**	0.30
Writing a novel	0.16	**0.80**
Writing a nonfiction book	**0.56**	0.33
Determining a person's creditworthiness	**0.67**	−0.14
Investing your pension fund's money	**0.75**	−0.17

*Note*: Factor loadings higher than 0.4 are shown in bold.

### 3.6 Trust and Confidence in AI

A second principal axis factor analysis was performed with the eight items on trust and confidence in AI. The scree plot and the eigenvalue criteria again suggested a two‐factor solution that explained 61.8% of the variance. Factor loadings after quartimax rotation with Kaiser normalization are reported in Table 4. While Factor 1 was named “AI‐trust,” Factor 2 was named “AI‐distrust.” Both factor scores were computed using the regression method.

**TABLE 4:** Rotated factor loadings for the items measuring trust and confidence in artificial intelligence (AI) (*N* = 923).

Rotated factor loadings	AI‐trust	AI‐distrust
AI can assess how good or bad its decisions were	**0.74**	0.06
AI can assess the consequences of its actions	**0.75**	0.03
AI makes fair decisions	**0.67**	−0.07
AI is perfect	**0.52**	−0.03
AI is trustworthy	**0.71**	−0.11
AI learns from its mistakes	**0.47**	−0.04
AI can intentionally do bad things	−0.12	**0.89**
AI can intentionally give wrong advice	−0.15	**0.92**

*Note*: Factor loadings higher than 0.4 are shown in bold.

### 3.7 Direct and Indirect Influence of General Confidence, General Trust, and Social Trust on Preferred Involvement of AI for Subjective and Objective Tasks

**FIGURE 3:**
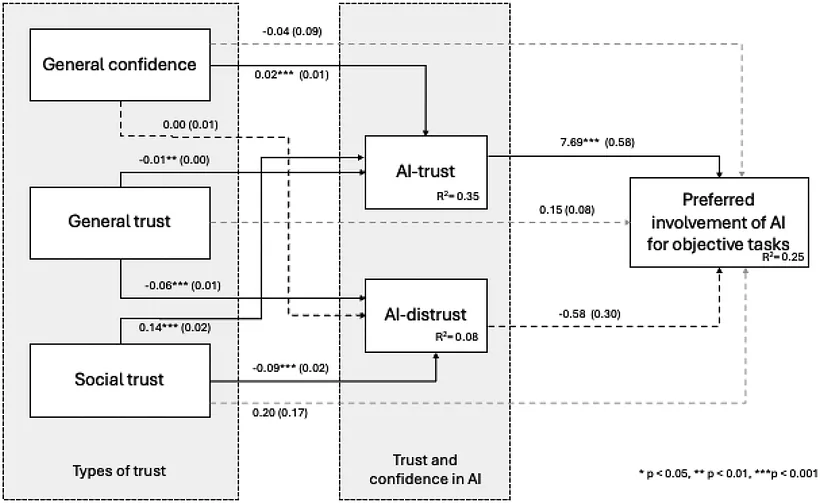
Unstandardized coefficients and standard errors for the model testing the influence of general confidence, general trust, and social trust on the preferred involvement of AI for objective tasks (*N* = 923). Solid lines indicate significant effects, dashed lines not significant effects. Postulated effects are black, not postulated effects are gray. *p < .05, **p < .01, ***p < .001.

**FIGURE 4:**
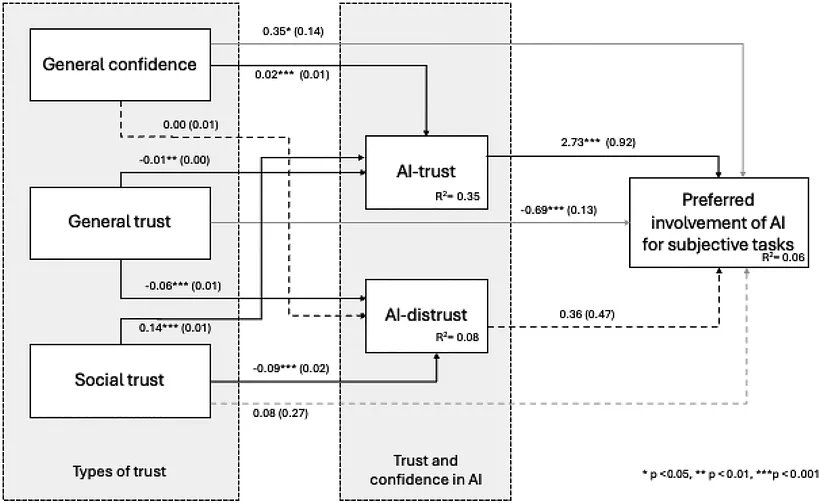
Unstandardized coefficients and standard errors for the model testing the influence of general confidence, general trust, and social trust on the preferred involvement of AI for subjective tasks (*N* = 923). Solid lines indicate significant effects, dashed lines not significant effects. Postulated effects are black, not postulated effects are gray. *p < .05, **p < .01, ***p < .001

Although we did not postulate any direct effects of general trust measures on the preferred involvement of AI for different tasks, for completeness, we nevertheless tested the direct and indirect influence of the different trust variables on the acceptance of AI. As the analysis of the factor structure of the different tasks indicated two factors, subjective and objective tasks, we decided to conduct the analysis for subjective and objective tasks separately. According to the procedure suggested by Hayes (2022), we used Model 4 with general confidence, general trust, as well as social trust as independent variables, and preferred involvement of AI as the dependent variable. The construct trust and confidence in AI was split into its two factors AI‐trust and AI‐distrust and included as mediators. Figures 3 and 4 show the tested models, including unstandardized coefficients and standard errors.

For objective tasks, general confidence, general trust, and social trust had significant indirect effects on the preferred involvement of AI. General confidence had a small positive influence on AI‐trust, but not on AI‐distrust. The effect of general trust on both AI‐trust and AI‐distrust was negative, but small. Social trust affected AI‐trust positively and AI‐distrust negatively. The direct effects of these general types of trust on the preferred involvement of AI for objective tasks were not significant. Although AI‐trust had a significant and positive effect on the acceptance of AI, the effect of AI‐distrust on the preferred involvement of AI for objective tasks was not significant. The model was able to explain 25% of the variance for the preferred involvement of AI for objective tasks.

For the subjective tasks, in addition to the indirect effect reported above, general confidence and general trust, but not social trust, had significant direct effects on the preferred involvement of AI. The preferred involvement of AI for subjective tasks was positively affected by AI‐trust, but not AI‐distrust. The model was able to explain 6% of the variance for subjective tasks.

## 4 Discussion

The present study examined the preferred involvement of AI for various tasks and investigated the influence of different types of trust on the preferred involvement of AI versus humans. The results show that the preferred involvement of AI varied between tasks. It was highest for “Searching for information,” “Making a weather forecast,” and “Programming software” where on average, participants preferred AI to be involved more than 60% and humans to less than 40%. In contrast to these three tasks, participants preferred the involvement of AI to be less than 30% for tasks like “Delivering a court judgment,” “Selecting a candidate for a job,” and “Driving a car.”

The findings of this study show that the preferred involvement of AI was higher for tasks that involve large amounts of data to process and that are typically performed with a computer such as searching for information, forecasting weather, or programming. It has been observed before that AI was perceived to be more suitable for tasks that require the processing and evaluation of data from multiple sources (Grassini et al. 2025). It is further in line with findings of Castelo et al. (2019) who showed that participants preferred algorithmic advice over human advice for weather forecasts and data analysis. These are tasks that typically cannot be performed without a computer.

Previous research also proposed that the degree of perceived subjectivity or objectivity influences consumers’ acceptance of AI for different tasks (Castelo et al. 2019). Tasks where only one correct solution is possible or where careful reasoning and systematic evaluation are required are considered objective tasks (Inbar et al. 2010; Logg et al. 2019). In line with the findings of Castelo et al. (2019), the present study also found perceived objectivity to be a significant predictor of the acceptance of AI. In addition, the ratings of preferred AI involvement showed an underlying factor structure that matched participants’ ratings of subjectivity versus objectivity.

Regarding our first research aim, which was to examine the qualitative characteristics of a task, we therefore summarize that the degree of perceived objectivity and the perceived level of risk in the event of errors influenced participants’ acceptance of AI for different tasks. These considerations likely influenced participants’ preferred AI involvement for tasks with far‐reaching consequences such as “Delivering a court judgment,” “Driving a car,” “Making a medical diagnosis,” and “Selecting a medical treatment” for which preferred AI involvement was lower. This finding is in line with previous research that found consumers to prefer humans over AI in the medical field (Kerstan et al. 2024; Larkin et al. 2021; Longoni et al. 2019; Promberger and Baron 2006). In the medical field, the low preferred AI involvement might not only be due to the fear of potential misdiagnosis and its consequences, but also due to perceived uniqueness neglect, which is the concern that individual characteristics are not sufficiently considered (Longoni et al. 2019; Mou et al. 2023).

The second research aim of this study was to assess the role of general trust‐related measures on trust and confidence in AI. The role of trust on the acceptance of AI has been explored before, and higher trust has been associated with higher acceptance of AI (Duan et al. 2025; Fahnenstich et al. 2024; Kerstan et al. 2024; Liu and Tao 2022; Liu et al. 2019; Mayer et al. 2024; Molina and Sundar 2024). It was, however, not clear which type of trust would be most important and whether different types of trust have opposing effects. In the present study, trust and confidence specifically in AI was measured with an ad hoc scale consisting of eight items assessing participants’ perceptions of AI's reliability and possible negative effects. The factor analysis revealed two components, which were named AI‐trust and AI‐distrust. Although the preferred involvement of AI for different tasks was positively influenced by AI‐trust, the effect of AI‐distrust was not significant. This indicates that participants with high AI‐trust had a higher preference for the involvement of AI for various tasks, which is in line with previous findings (Duan et al. 2025; Fahnenstich et al. 2024; Kerstan et al. 2024; Liu and Tao 2022; Liu et al. 2019; Mayer et al. 2024; Molina and Sundar 2024). Although it was expected that the direct measure of AI trust and confidence would influence the preferred involvement of AI for different tasks strongly, it was interesting to see that the effect of AI‐trust on the preferred involvement of AI for subjective and objective tasks was rather large, but that AI‐distrust did not influence the preferred involvement of AI. A possible explanation for this might be that the preferred level of AI‐involvement is driven by competence rather than (bad) intentions. This seems plausible as the items for measuring AI‐trust were competence‐oriented and AI‐trust significantly influenced preferred involvement of AI for different tasks. It is further worth noting that the three general trust‐related measures, general confidence, general trust, and social trust, had an indirect influence on the preferred involvement of AI through AI‐trust.

For general confidence, we had expected it to have a positive influence on trust and confidence in AI. People with high general confidence trust that events will occur as expected and that society is equipped to cope with future problems and risks (Keller et al. 2011). That people with high general confidence would be more skeptical toward AI for solving problems compared to people with low general confidence was thus expected. For both types of tasks, this expectation could, however, only be partly confirmed, as general confidence was positively associated with AI‐trust, but not with AI‐distrust.

Regarding general trust, we had proposed that it would negatively affect trust and confidence in AI. We expected this relationship to be negative as we assumed that individuals who generally believe in the goodwill and honesty of others would be more wary against AI taking over tasks from humans. For subjective and objective tasks, we found that general trust was negatively associated with AI‐trust and AI‐distrust. Although the negative relationship of general trust on AI‐trust was weaker than expected, it is in line with previous research that has shown that people with high general trust sometimes perceive less risk, for example, in the case of the coronavirus (Siegrist, Luchsinger, et al. 2021). The also negative impact of general trust on AI‐distrust was surprising at first but might be explained by participants’ interpretation of the distrust items in the way that humans are programming AI to intentionally do bad things and give wrong advice. Participants with high general trust might believe that humans are generally trustworthy and therefore also less likely to program AI to intentionally do bad things.

Finally, our hypothesis that social trust would positively influence people's trust and confidence in AI was confirmed. Social trust was positively associated with AI‐trust and negatively associated with AI‐distrust. Social trust was not directly associated with the preferred involvement of AI for the different tasks. Nevertheless, social trust often serves as a heuristic when people evaluate the risks and acceptability of a technology (Siegrist 2021). This is reflected in our findings as individuals who have higher trust in tech companies and in authorities regulating AI show higher AI‐trust and lower AI‐distrust. This observation is further in line with previous results from Liu et al. (2019) showing that social trust not only influences perceived benefits and risks, but also positively influences acceptance of AI for automated driving.

Castelo et al. (2019) found that algorithms were less trusted for subjective tasks compared to objective tasks. Our study indicates that different kinds of trust have a similar effect on the evaluation of both objective and subjective tasks. This seems counterintuitive as it contradicts the notion that trust matters most when individuals cannot rely on their own expertise (Siegrist and Cvetkovich 2000). For subjective tasks (composing a song, writing a novel), the individual is able to judge whether the AI (or another human) performed well. One either likes the result or one does not. In addition, the consequences of making a wrong judgment are extremely small as it is relatively easy to stop listening to a song or give up reading a book. For objective tasks, however, the situation is different. Unless highly knowledgeable in the particular field, most individuals are unable to accurately evaluate the quality of a decision for an objective task like making a medical diagnosis, driving a car, or delivering a court judgment. Here, individuals rely on the assessment of an expert. As a result, the perceived quality of such a decision is largely determined by the extent to which individuals trust the experts involved.

Cross‐cultural differences in social trust, general trust, and confidence, as well as exposure to AI technology and privacy concerns, may influence how consumers perceive and accept AI for different tasks. This might limit the generalizability of our findings for other cultures. Previous research in cultural psychology suggests that it is not geographical distance, but rather the role of cultural identity that explains differences in the acceptance of AI (Barnes et al. 2024). Individualistic cultures (e.g., Switzerland) are more likely to view AI as external to the self and therefore perceive it as a threat to their uniqueness, autonomy, and privacy. Conversely, collectivistic cultures (e.g., China) are more likely to see AI as an extension of the self and may therefore see AI as a means of conforming to consensus and protecting privacy (Barnes et al. 2024).

To summarize, the present study showed that different types of trust influenced consumers’ preferred involvement of AI. The preferred involvement of AI for objective and subjective tasks was largely and directly influenced by AI‐trust, but not AI‐distrust and indirectly affected by general confidence, general trust, as well as social trust. On average, participants were comfortable with AI to be involved to a degree of at least 20% in all tasks. When it came to searching for information, predicting the weather, or programming software, participants even indicated that they were comfortable with AI being involved around 60%, meaning that AI would take over more than half of the task. As AI has the potential to improve the fulfillment of many tasks, but even the most beneficial AI application will fail if it is not accepted by consumers, further increasing trust is a promising strategy to increase AI acceptance. This approach is particularly relevant because trust not only affects acceptance directly but also indirectly through general confidence, general trust, and social trust.

## 5 Limitations

Although the present work provides valuable insight, it does not come without limitations. First, our results represent a single time point measurement. Given the ongoing evolution of AI technology and the expansion of its applications, consumer attitudes are likely to change with increasing use and knowledge of the technology. Second, referring to “AI” for all the different applications involved in the presented tasks represents a limitation as we treated it as a single, homogenous construct. In reality, AI is an umbrella term and covers different subfields such as machine learning, natural language processing, computer vision, or robotics. Simply calling it AI may not have adequately addressed the nuances, which could technically make a difference for the acceptance. However, we considered this simplification justified as consumers in everyday language do not distinguish between the different applications (except maybe ChatGPT) but rather call it AI. Third, it might have been difficult for consumers to evaluate the tasks as we did not describe how AI exactly would be involved and what current AI applications are able to do and what not. This uncertainty might have negatively or positively influenced their answers.

Fourth, although we provided a framework of plausible reasons why and how the different trust measures affect the preferred involvement of AI for different tasks, the mediation model is based on correlational data. It can therefore not be ruled out that there are other models that would also fit the correlations. Further, correlations do not equate to causation even though mediation analysis per definition assumes a causal chain of events.

Finally, Switzerland is a highly individualistic culture that may be more likely to perceive AI as a potential threat to personal uniqueness, autonomy, and privacy (Barnes et al. 2024). For future research, we therefore recommend examining different cultures. As our selection of tasks turned out to include more tasks that were perceived as objective compared to subjective, future research may also include more tasks that are likely to be perceived as subjective such as “Giving a fashion style recommendation” or “Suggesting a decoration for the living room.”

## 6 Conclusion

Participant's preferred involvement of AI was highest for the tasks “Searching for information,” “Making a weather forecast,” and “Programming software” and lowest for “Delivering a court judgment,” “Selecting a candidate for a job,” and “Driving a car.” Perceived objectivity emerged as positive and perceived level of risk in the event of errors as negative significant predictors for participants’ preferred involvement of AI. Further, the preferred involvement of AI for objective and subjective tasks was largely and positively influenced by AI‐trust but not AI‐distrust. General confidence, general trust, and social trust had indirect and smaller effects on the preferred involvement of AI for different tasks. These findings emphasize that trust is a key factor for the acceptance of AI as even the most helpful AI application will not be successful unless consumers trust these applications.

## Author Contributions

**Fabienne Michel**: conceptualization, methodology, formal analysis, investigation, writing – original draft, visualization. **Michael Siegrist**: conceptualization, methodology, writing – original draft, review and editing.

## Funding

The authors have nothing to report.

## Ethics Statement

The study was approved by ETH Zurich Ethics Commission on March 13, 2025 (proposal number: 25 ETHICS‐061).

## Conflicts of Interest

The authors declare no conflicts of interest.

## Data Availability

The data will be made available upon request.
